# 2227. Assessing Clinicians' and Trainees' Knowledge and Practice of the IDSA Asymptomatic Bacteriuria Guidelines in the Elderly

**DOI:** 10.1093/ofid/ofad500.1849

**Published:** 2023-11-27

**Authors:** Eleanor E A Smith, Nagakrishnal Nachimuthu, Jose A Negrete, John Flynn, Mayar Al Mohajer

**Affiliations:** Baylor College of Medicine, Houston, Texas; Baylor College of Medicine, Houston, Texas; Baylor College of Medicine, Houston, Texas; CommonSpirit Health Texas Division, Houston, Texas; Baylor College of Medicine, Houston, Texas

## Abstract

**Background:**

Asymptomatic bacteriuria (ASB) is common in the elderly; however, its treatment in this population is not beneficial. Older individuals are also more susceptible to the adverse effects of antibiotics. In practice, many older patients with ASB and new delirium or a fall are empirically treated for presumed UTI, despite a 2019 update to the Infectious Disease Society of America (IDSA) ASB guidelines that recommends against this practice. We aimed to identify self-reported familiarity with the IDSA ASB guidelines, gaps in knowledge, and cognitive-behavioral constructs (CBCs) relevant to treating ASB in older patients.

**Methods:**

This cross-sectional study included advanced practitioners, physicians, residents, and medical students at one academic medical center with affiliated community practices in Southeast Texas. Compared to previous studies, this sample included a greater variety of practitioners from both academic and community settings. The main outcomes were self-reported guidelines familiarity, knowledge score, and behavior score. Chi-squared, one-way ANOVA, and two multivariable linear regressions were performed to assess the relationship between outcomes and categorical variables.

**Results:**

154 out of 1,305 participants (11.8%) responded. The mean knowledge score was 64.87 (SD 23.65), and the mean behavior score was 2.73 (SD 0.91, Table 1). Higher knowledge scores were significantly associated with better antibiotic stewardship practices (P< 0.001) and a better understanding of risks associated with treating ASB (P=0.019, Table 2). Self-reported familiarity with the guidelines did not correlate with knowledge scores (P=0.807). Higher behavior scores were significantly associated with better familiarity (P=0.002), workplace social norms (P< 0.001), knowledge scores (P< 0.001), and risk perceptions (P< 0.001).

**Figure:**
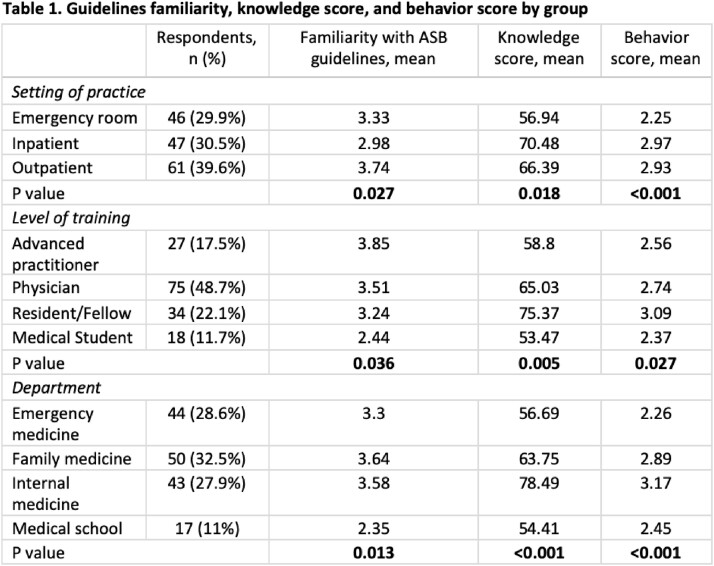

**Figure:**
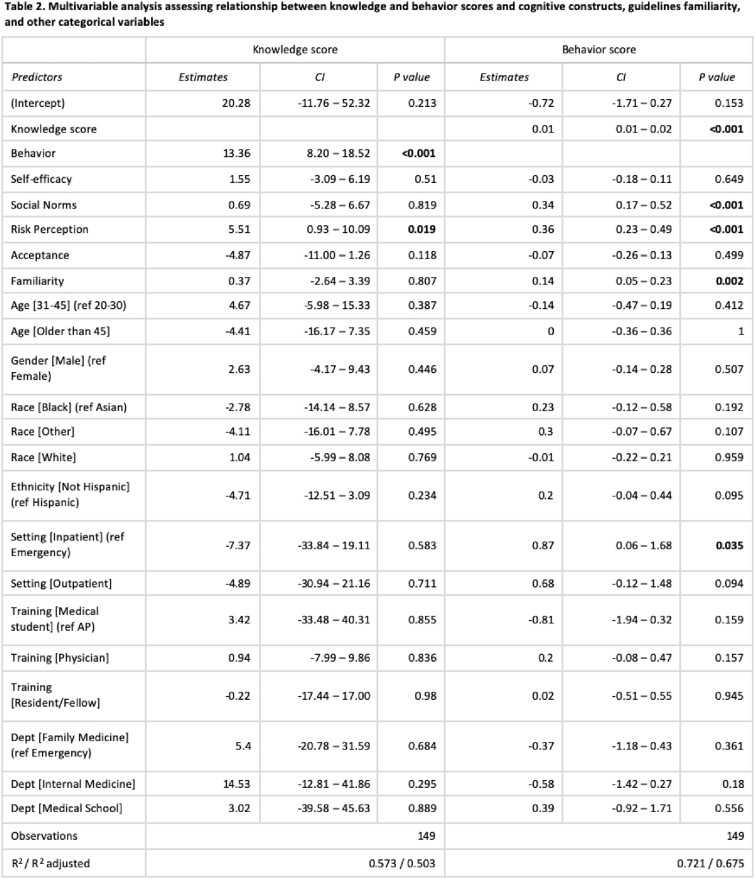

Multivariable analysis assessing relationship between knowledge and behavior scores and cognitive constructs, guidelines familiarity, and other categorical variables

**Conclusion:**

There is a gap in knowledge and practice related to antibiotic use for ASB in elderly patients. To optimize antibiotic stewardship, efforts should focus on improving knowledge, familiarity with guidelines, social norms, and risk perceptions.

**Disclosures:**

**All Authors**: No reported disclosures

